# Topical ocular delivery of nanoparticles with epoetin beta in Wistar Hannover rats

**DOI:** 10.1038/s41598-023-28845-0

**Published:** 2023-01-27

**Authors:** Beatriz Silva, Lídia M. Gonçalves, Berta São Braz, Esmeralda Delgado

**Affiliations:** 1 CIISA-Centre for Interdisciplinary Research in Animal Health, Faculty of Veterinary Medicine, Universidade de Lisboa, Avenida da Universidade Técnica, 1300-477 Lisbon, Portugal; 2 Associate Laboratory for Animal and Veterinary Sciences (AL4AnimalS), 1300-477 Lisbon, Portugal; 3 Research Institute for Medicines (iMed.ULisboa), Faculty of Pharmacy, Universidade de Lisboa, 1649-003 Lisbon, Portugal

**Keywords:** Drug discovery, Medical research

## Abstract

Topical instillation of drugs targeting the posterior ocular segment is an expanding area of research. Chitosan and hyaluronic acid have remarkable mucoadhesive properties and potentially enhance pre-corneal retention time after topical instillation. Bearing this in mind, we explored the possibility of delivering epoetin beta (EPOβ) to the posterior segment of the eye in a chitosan-hyaluronic acid (CS/HA-EPOβ) nanoparticulate system using the topical route of administration. Complete ophthalmological examinations, electroretinography and microhematocrit evaluations were performed in Wistar Hannover (WH) rats, before and after topical administration of nanoparticles. The right eye received CS/HA-EPOβ and the left eye received only empty nanocarriers (control). Animals were split into 6 groups and at designated timepoints, all animals from each group (n = 3) were euthanized and both eyes enucleated. Retinal morphology and EPOβ ocular distribution were assessed, respectively, through hematoxylin and eosin (HE) and immunofluorescence staining. After topical administration, no adverse ocular signs were noted and no significant changes either in microhematocrits nor in electroretinographies were detected. During the study, intraocular pressure (IOP) was always kept within physiological range bilaterally. No histological changes were detected in any of the ocular globes. Immunofluorescence enabled the identification of EPOβ in the retina 12 h after the administration, its presence still being detectable at day 21. In conclusion, CS/HA nanoparticles could efficiently deliver EPOβ to the retina of WH rats after topical instillation, being considered biologically safe. Topical administration of this nanoformulation could be a valuable tool for retinal neuroprotection, decreasing risks associated with more invasive routes of administration, being cost effective and also increasing long-term patients’ compliance.

## Introduction

Glaucoma is a chronic neurodegenerative disease, being one of the world’s leading causes of blindness. The use of neuroprotective drugs targeting the retina as part of the glaucoma treatment has been a compelling field of study^1^. It is known that erythropoietin (EPO) acts as a protective agent in organs and tissues such as the brain, heart, inner ear and retina, and the recombinant forms of EPO, like epoetin beta (EPOβ), are currently of great interest in ophthalmology research^2^. EPOβ has revealed beneficial effects in the retina of rats with glaucoma after subconjunctival administration^3^.

Despite the crucial need for alternative therapeutics, efforts to develop novel glaucoma treatments have met with limited success. In an attempt to address this issue, our team recently developed and characterized a chitosan-hyaluronic acid nanoparticulate system conceived to carry EPOβ into the ocular globe, and performed in vitro and ex vivo tests to ensure its physicochemical stability, mucoadhesive strength and safety^4^. The ex vivo permeation in porcine conjunctiva, sclera and cornea, revealed that EPOβ was able to trespass the three ocular membranes, which supported the in vivo investigation of topical ocular delivery of these nanocarriers^4^. Moreover, we tested this nanoformulation in Wistar Hannover rats using subconjunctival administration, confirming its biological safety and EPOβ delivery to the retina^5^. Therefore, we considered the topical ocular administration of EPOβ in chitosan-hyaluronic acid nanoparticles to be a very interesting approach, because it has never been tested before and merged the advantages of enhancing corneal and conjunctival contact time, of being user friendly, cost effective and potentially leading to fewer side effects than other routes of administration. When considering the treatment of posterior ocular segment diseases, intravitreal, systemic, and subconjunctival routes of administration are usual choices. However, intravitreal injection is an invasive procedure and it has several potential side effects like intraocular hemorrhage, endophthalmitis, cataracts, vitreous detachment, increased intraocular pressure and retinal toxicity^6^. Subconjunctival administration is less invasive and has minor side effects^7^ but patients cooperation and/or a light sedation are still required. Systemic administration demands high drug doses which may result in critical secondary effects^6^. Therefore, despite mechanisms like blinking, nasolacrimal drainage and tear turnover, that hinder drugs permeation through ocular tissues after topical administration, the development of topical instillation of drugs targeting the posterior ocular segment is an expanding area of research^8,9^.

Mucoadhesive nanoparticles are a way of enhancing drugs permeation across biological ocular barriers, while protecting them from the ocular environment, increasing drugs intraocular concentration and bioavailability. They play an important role as vehicles in topical ocular administration, due to their enhanced connection with the ocular mucosa^10^. Chitosan is a mucoadhesive natural polymer with cationic nature, that establishes ionic interactions with the negatively charged ocular mucosa and also widens the tight junctions of the cell membranes^9,11–13^. Recent studies using chitosan based nanoparticles, applied by topical route of administration in rats and rabbits, observed a greater permanence of the nanoparticles on the ocular surface^14,15^, and also a higher therapeutic activity using anti-inflammatory and antimicrobial drugs, when compared to the control formulations^16,17^. Thus, chitosan can increase drugs pre-corneal retention time, which might reduce the frequency of the administration and improve patients compliance^11,12,18^. In addition, chitosan mucoadhesiveness can be enhanced by association with hyaluronic acid, which is a natural polymer found in ocular tissues like vitreous, lacrimal gland, conjunctiva, corneal epithelium and also in human tears^19^. Hyaluronic acid is widely used in ophthalmic formulations due to its viscoelasticity and mucoadhesiveness associated to CD44 receptors located in corneal epithelium and endothelium^20–22^. Being so, after topical administration, chitosan-hyaluronic acid nanoparticles are able to improve drugs retention time on ocular surface and their penetration through the ocular tissues, potentially increasing their bioavailability^18^.

As mentioned, the nanoparticulate system of chitosan-hyaluronic acid-epoetin beta (CS/HA-EPOβ) developed by our team underwent previous in vitro, ex vivo^4^, and subconjunctival in vivo tests^5^. In the present study, we aimed to explore the in vivo biological impact of this nanoformulation in healthy Wistar Hanover rats using the topical ocular route of administration. We proposed to evaluate the topical tolerance, safety, systemic and local impacts, the effect in retinal morphology and electrophysiology, and EPOβ ocular distribution using immunohistochemistry. Our team is committed to pursuit this promising line of research by using CS/HA-EPOβ nanoparticles as a non-invasive neuroprotective and neuroregenerative co-adjuvant treatment targeting the retina of glaucomatous animals.

## Results

### Ophthalmological examinations

All animals showed very good tolerance to the CS/HA nanoparticles, either loaded with EPOβ (OD) or empty, that is, without EPOβ (OS). After the instillation of the nanoformulation, no signs of discomfort or pain were observed, and all animals exhibited normal behavior throughout the entire study. No signs of stress were observed, like obsessive–compulsive behavior, apathy, alopecia or other skin lesions. No discrepancies in food and water intake, nor abnormal changes in body weight were detected. Likewise, no abnormal ocular signs were observed, such as pruritus, epiphora, blepharospasm, conjunctival hyperemia, corneal edema, ulcerations, keratitis, etc. Therefore, ophthalmological examinations of both eyes were considered normal for all groups (n = 18) throughout the study after topical administration of the nanoparticles.

**Figure 1:**
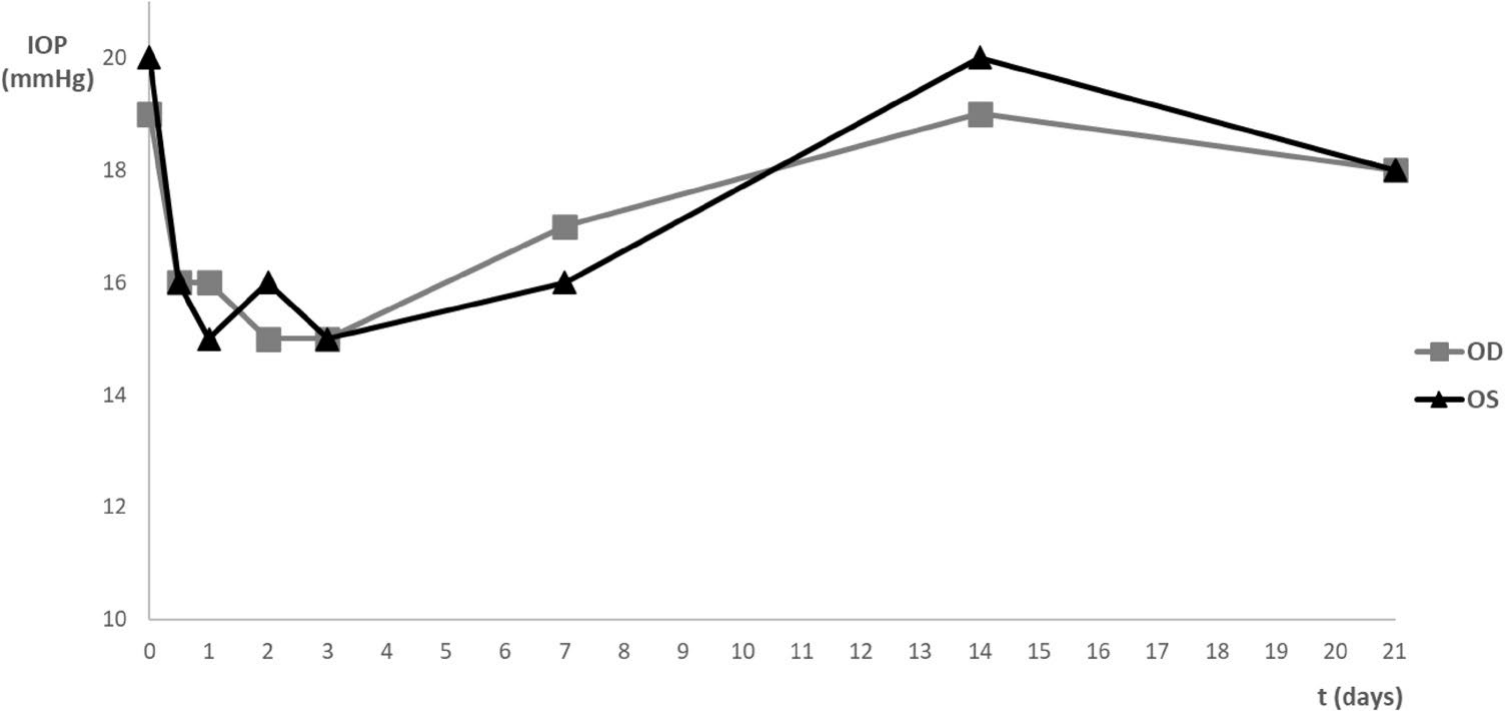
Variation of the mean IOP (mmHg) through time after topical administration of CS/HA-EPOβ loaded nanoparticles to the right eye (OD) and empty CS/HA nanoparticles to the left eye (OS). Values represent the mean values of all groups, in the right eye (OD) and in the left eye (OS). [Microsoft Excel (2018). Microsoft Corporation, Redmond, Washington, USA. https://office.microsoft.com/excel].

Regarding the intraocular pressure (IOP), mean values for the OD and the OS before the topical administration were 18 ± 1 mmHg and 19 ± 2 mmHg, respectively. Figure 1 represent the mean IOP variation of both eyes after topical instillation of the nanocarriers. There were statistically significant differences (*p* < 0.05) between the mean IOP immediately after the administration (t = 0) and the IOP of the following days, from 12 h (t = 0.5) to day 7 (t = 7), with an average variation of 5 mmHg. These differences were no longer evident at day 14 and 2. Comparing the OD (treatment) and the OS (control), no statistically significant differences (*p* > 0.05) were detected between the mean IOP of both eyes in any of the groups/ timepoints, meaning that the CS/HA nanoparticles, with and without EPOβ, seemed to not influence the ocular physiology.

### Hematocrit

Rats’ hematocrits were stable during the entire study. The mean microhematocrit values before and after topical administration of the CS/HA-EPOβ nanoparticles were 45.3 ± 2.5% and 45.2 ± 2.8%, respectively. No significant variations in the microhematocrit values were detected between results obtained before and after topical administration (*p* > 0.05), which means that the impact of CS/HA-EPOβ nanoparticles in erythropoiesis was inconsiderable.

### Electroretinography

**Figure 2:**
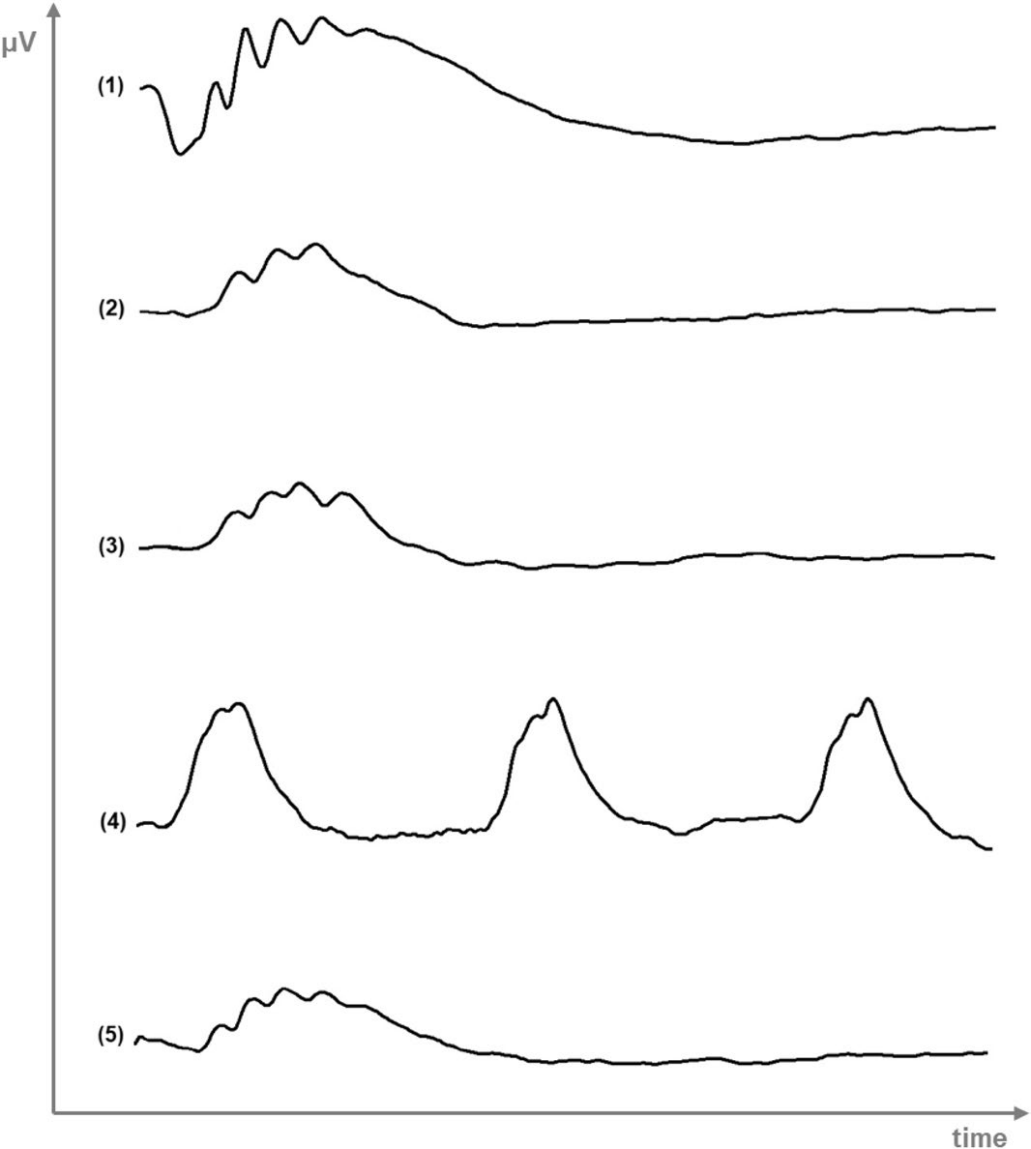
Example of an ERG trace from a rat, showing the waveforms recorded in the different phases of the protocol: (1) scotopic luminesce response at 5 dB; (2) photopic adaptation at 16 min; (3) photopic luminescence response at 5 dB; (4) photopic flicker at 0 dB; (5) scotopic adaptation at 32 min. [Microsoft Paint 3D (2018). Microsoft Corporation, Redmond, Washington, USA].

**Figure 3:**
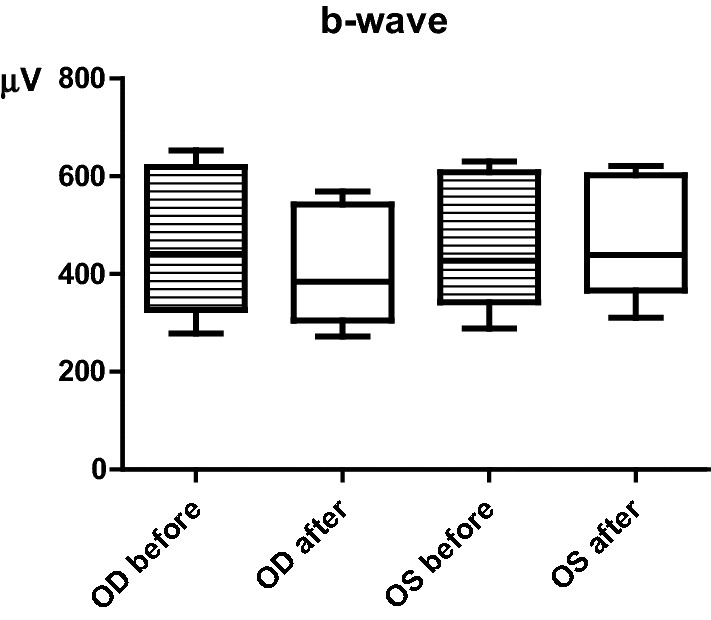
Mean amplitudes of the b-wave recorded from the OD and OS in the SLR, before and after topical administration of the nanoparticles (after = immediately before euthanasia). Data is presented in µV as mean ± SD and represents all groups. [GraphPad—GraphPad Prism version 8.0.0 for Windows, GraphPad Software, San Diego, California USA, www.graphpad.com].

Flash ERG essentially records rods and cones activity as a-wave and b-wave amplitudes (μV) in response to different intensities and frequencies of luminous stimuli. It was performed to evaluate the retinal response to the CS/HA-EPOβ nanoparticulate system. Figure 2 is a representation of an ERG trace recorded from a rat in this study, whereas mean results are detailed described below as mean ± SD [min; max] μV (Fig. 3).

In the scotopic luminescence response (SLR), the amplitudes of both a and b waves increased with light intensity. Before CS/HA-EPOβ administration, at –35 dB of light (–3.02 log cds/m^2^) the a-wave was 22 ± 9 [3; 43] μV for the OD and 25 ± 17 [3; 64] μV for the OS; and the b-wave amplitude was 278 ± 83 [137; 471] μV for the OD and 288 ± 87 [151; 445] μV for the OS. At a light intensity of 5 dB (0.98 log cds/m^2^), the a-wave was 268 ± 67 [159; 356] μV for the OD and 263 ± 47 [173; 323] μV for the OS; while the b-wave was 653 ± 128 [349; 858] μV for the OD and 628 ± 83 [478; 763] μV for the OS. Both a and b waves mean amplitudes, before and after the topical administration, were not significantly different (*p* > 0.05) between both eyes and among groups. For instance, after the topical administration, at 5 dB, the mean a-wave for the OD was 215 ± 79 [114; 375] μV and the b-wave was 585 ± 113 [443; 789] μV. Additionally, the OS showed a mean a-wave of 247 ± 57 [159; 326] μV and a b-wave of 612 ± 103 [399; 766] μV. Figure 3 illustrates the mean b-wave amplitudes for both the OD and the OS, considering the nine different light intensities of the SLR.

No statistically significant differences (*p* > 0.05) were observed in the photopic adaptation period (PA) between treated (OD) and control (OS) eyes, and there were also no substantive changes among groups. At the end of this phase (16 min of light adaptation), before the topical administration, the OD presented mean a and b waves of 18 ± 16 [2;61] μV and 220 ± 52 [135; 312] μV, respectively. The OS had mean a-waves of 17 ± 15 [1;54] μV and b-waves of 240 ± 59 [164; 357] μV. After the topical administration, the mean a-wave was 15 ± 12 [1; 39] μV for the OD and 17 ± 16 [2; 66] μV for the OS; while the b-wave was 200 ± 35 [145; 254] μV for the OD and 224 ± 49 [153; 357] μV for the OS. Results in Table 1 correspond to the mean waves values considering the five steps of the light adaptation period.

**Table 1:** Representation of the a (a) and b (b) waves (µV; mean ± SD) recorded from the OD and the OS during the five steps of the PA, before and after the topical administration of the nanoparticles (after = immediately before euthanasia). Results correspond to the sum of the results from all groups.

Minutes	b-wave (µV)				a-wave (µV)				
	OD		OS		OD			OS	
	Before	After	Before	After	Before		After	Before	After
0	226 ± 36	218 ± 44	225 ± 45	249 ± 69	21 ± 16		18 ± 15	20 ± 19	15 ± 11
2	200 ± 38	191 ± 32	209 ± 46	121 ± 42	14 ± 11		17 ± 15	24 ± 24	21 ± 12
4	211 ± 54	198 ± 36	215 ± 43	78 ± 33	18 ± 12		20 ± 15	16 ± 12	15 ± 12
8	222 ± 46	196 ± 38	235 ± 47	66 ± 42	22 ± 23		17 ± 14	20 ± 18	20 ± 17
16	220 ± 52	200 ± 35	240 ± 59	65 ± 38	18 ± 16		15 ± 12	17 ± 15	17 ± 16

**Figure 4:**
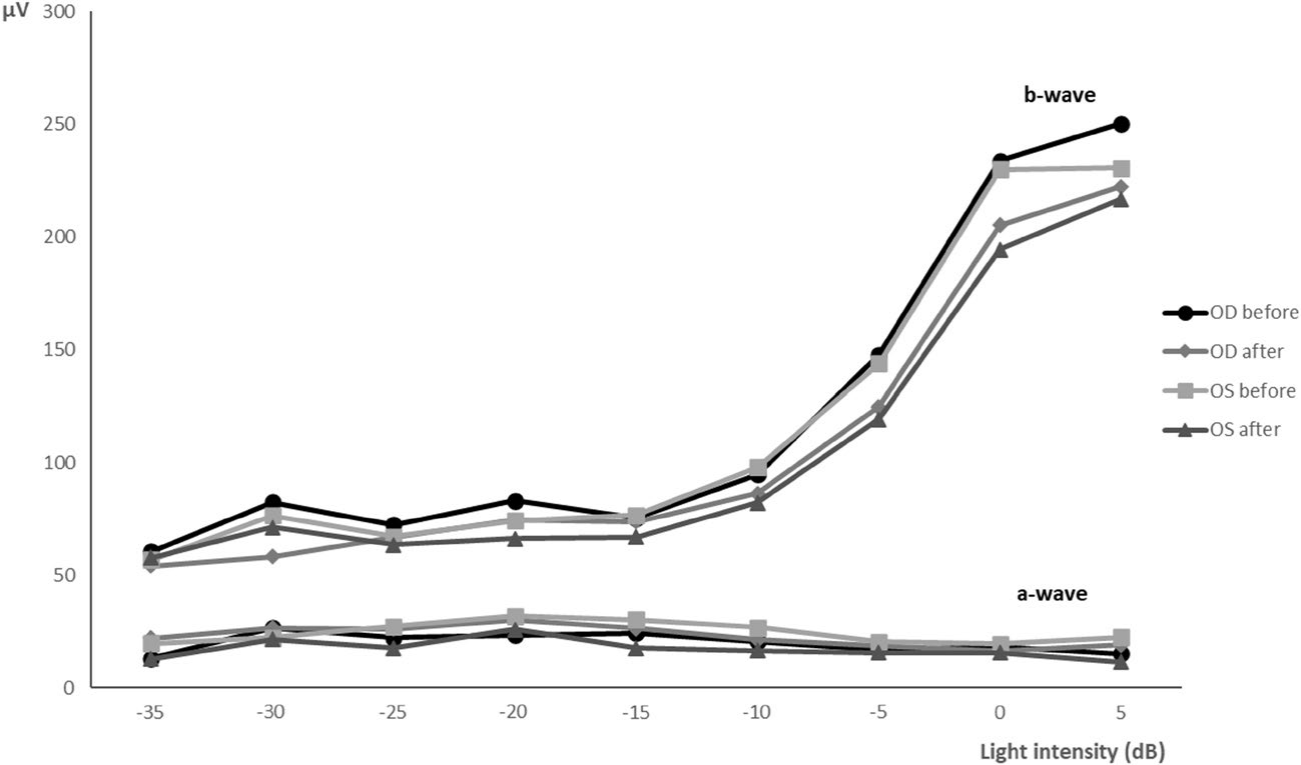
Representation of the a and b waves mean amplitudes recorded from the OD and OS during the PLR, before and after the topical administration of the nanoparticles (after = immediately before euthanasia). Data represent all groups (µV; mean ± SD). [Microsoft Excel (2018). Microsoft Corporation, Redmond, Washington, USA. https://office.microsoft.com/excel].

Photopic luminescence response (PLR) results show that the b-wave amplitude increases with light intensity, while the a-wave amplitude was nearly constant (Fig. 4). No statistically significant differences (*p* > 0.05) were noticed among groups or between OD and OS, as the mean amplitudes of the a-waves and b-waves were comparable before and after the topical administration of the nanoparticles. At 5 dB of light intensity, the OD showed a mean a-wave of 15 ± 14 [2; 51] μV and a b-wave of 250 ± 40 [186; 323] μV before the administration; and a-wave of 16 ± 14 [2; 58] μV and a b-wave of 222 ± 52 [124; 371] μV after the administration. On the other hand, before the administration, the OS presented a mean a-wave of 18 ± 17 [1; 60] μV and a b-wave of 231 ± 61 [99; 369] μV; and after the administration, the a-wave and b-wave were, respectively, 14 ± 12 [4; 49] μV and 218 ± 47 [111; 304] μV.

Photopic flicker (PF) results are presented in Table 2, which show no statistically significant differences (*p* > 0.05) between OD and OS, before or after topical administration. The a and b waves recorded at 0 db, before the administration were, respectively, 6 ± 3 [2; 14] μV and 236 ± 55 [108; 348] μV for the OD; and 7 ± 3 [3; 15] μV and 231 ± 36 [164; 294] μV for the OS. After the administration, the a and b waves were, respectively, 5 ± 4 [1; 18] μV and 230 ± 48 [148; 354] μV for the OD; and 7 ± 4 [2; 18] μV and 249 ± 69 [139; 387] μV for the OS.

**Table 2:** Mean amplitudes of the a-wave and b-wave (µV; mean ± SD) recorded from the OD and OS in the PF, in response to decrescent light-stimuli (0, –5, –10 and –15 dB).

Light (dB)	b-wave (µV)				a-wave (µV)				
	OD		OS		OD			OS	
	Before	After	Before	After	Before		After	Before	After
0	236 ± 55	230 ± 48	231 ± 36	249 ± 69	6 ± 3		5 ± 4	7 ± 3	7 ± 4
–5	127 ± 38	135 ± 52	130 ± 36	121 ± 42	18 ± 15		16 ± 22	15 ± 11	18 ± 15
–10	73 ± 19	76 ± 38	80 ± 27	78 ± 33	18 ± 14		13 ± 13	14 ± 8	14 ± 9
–15	65 ± 38	59 ± 45	74 ± 53	66 ± 42	19 ± 11		16 ± 12	14 ± 8	20 ± 13

Values correspond to before and after administration of nanoparticles (after = immediately before euthanasia). Data represents all groups.

Table 3 represents the scotopic adaptation (SA) results, which is the last step of the ERG exam. In all groups, both a and b waves showed an increase in their amplitudes (μV) after the dark adaptation period. At 32 min of dark adaptation, before the topical administration, the mean a-wave was 91 ± 43 [34; 207] μV for the OD and 87 ± 31 [46; 140] μV for the OS; while the mean b-wave was 395 ± 109 [277; 685] μV for the OD and 414 ± 67 [293; 541] μV for the OS. After the administration, the a and b wave were, respectively, 109 ± 43 [34; 198] μV and 455 ± 51 [339; 553] μV for the OD and 100 ± 36 [53; 170] μV and 421 ± 74 [241; 532] μV for the OS. Likewise, results show no significant differences (*p* > 0.05) between the period before and after the administration of the nanoparticles, and between the OD and the OS, in any of the groups.

**Table 3:** a and b waves mean amplitudes (µV) recorded from the OD and the OS in the SA, after 0 to 32 min of dark adaptation.

Minutes	b-wave (µV)				a-wave (µV)				
	OD		OS		OD			OS	
	Before	After	Before	After	Before		After	Before	After
0	270 ± 65	258 ± 61	281 ± 76	237 ± 46	28 ± 30		30 ± 22	27 ± 17	28 ± 15
2	285 ± 74	278 ± 74	285 ± 69	284 ± 73	47 ± 34		43 ± 23	45 ± 25	41 ± 19
4	299 ± 75	308 ± 73	303 ± 61	291 ± 56	48 ± 32		58 ± 29	40 ± 17	46 ± 22
8	336 ± 78	337 ± 59	350 ± 58	322 ± 55	60 ± 38		62 ± 27	63 ± 31	55 ± 25
16	353 ± 87	391 ± 49	374 ± 71	361 ± 73	71 ± 34		80 ± 40	70 ± 32	77 ± 32
32	395 ± 109	455 ± 51	414 ± 67	421 ± 74	91 ± 43		109 ± 43	87 ± 31	100 ± 36

Data represents all groups, before and after administration of the nanocarriers (after = immediately before euthanasia).

Considering the results from the five steps of the ERG exam, the CS/HA nanoparticles, with and without encapsulated EPOβ, did not cause any adverse side effects in the electrical retinal activity when applied topically, since no statistically significant differences were observed in the retinal response between the OD and the OS in any of the group (from A to F), and in any step of the ERG exam (*p* > 0.05). Moreover, results before and after the administration of the nanoparticles were similar for both eyes (*p* > 0.05).

### Histological and immunohistochemistry evaluation

**Figure 5:**
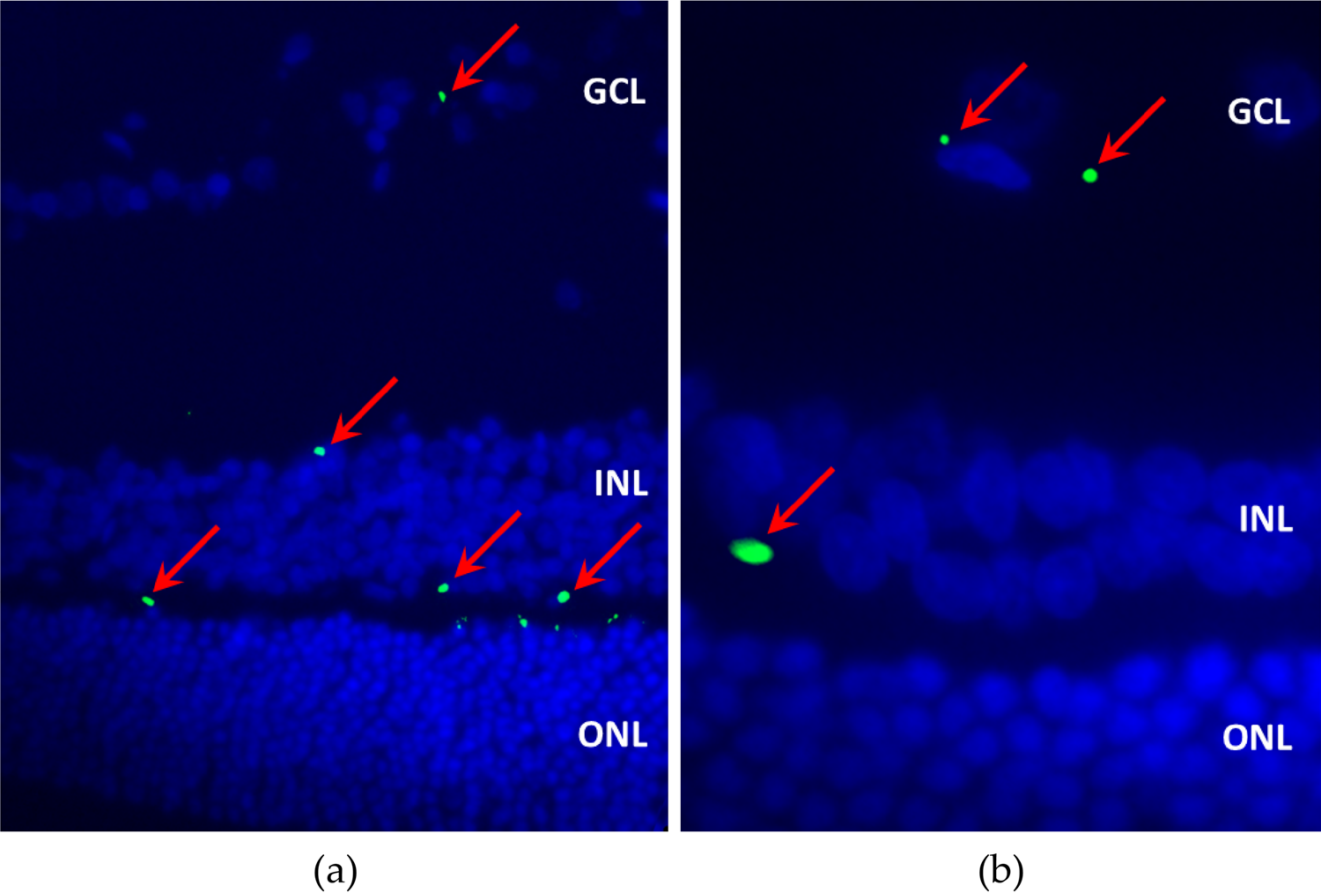
Immunofluorescence images showing cross sections of the retina after CS/HA-EPOβ topical administration: (**a**) OD from group B (magnification $\times$40); (**b**) OD from group E (magnification $\times$100). Images show the merging of the green and the blue filters. Red arrows indicate EPOβ presence (green), while cell nuclei are stained in blue by DAPI. GCL, ganglion cell layer; INL, inner nuclear layer; ONL, outer nuclear layer. [Images edited in ImageJ (Rasband, W.S., ImageJ, U. S. National Institutes of Health, Bethesda, Maryland, USA, https://imagej.nih.gov/ij/, 1997–2018. Schneider, C.A., Rasband, W.S., Eliceiri, K.W.) and Microsoft Paint 3D (2018). Microsoft Corporation, Redmond, Washington, USA.)].

At the end of this study, immunofluorescence was performed to evaluate EPOβ’s distribution throughout the ocular globe and the hematoxylin and eosin (HE) staining was used to assess cellular structure of the ocular tissues, especially retinal morphology. Immunofluorescence results showed that EPOβ was detected in the retina, more precisely at the retinal ganglion cell layer, of the OD of all animals from group A, which corresponded to 12 h after topical administration of the nanoformulation. At that timepoint, EPOβ was also observed in the corneal stroma, corneal endothelium, ciliary body, posterior capsule of the lens, vitreous and sclera of the OD. In the remaining timepoints, EPOβ was mostly detected in the different retinal cell layers, followed by vitreous, choroid and sclera. In group E (14 days), EPOβ was still detected in the corneal stroma, denoting a sustained transcorneal permeation. The intensity and amount of fluorescent dots (EPOβ) declined with time, and at day 21 (group F) only fluorescent remnants were observed in the retina. Figure 5 shows EPOβ distributed throughout the retinal layers of group B and E. No EPOβ was observed in any of the control eyes (OS).

Regarding the HE staining, cross sections of the ocular globes were analyzed in terms of cellular damage and alterations in cell/ tissue morphology, after topical administration of the nanoparticles. No histological changes were observed in any of the ocular globes, and all groups presented comparable characteristics between the OD and the OS, meaning that the administration of the CS/HA nanoparticles, with and without EPOβ, seemed innocuous to retinal structure.

## Discussion

In the past, erythropoietin was considered solely an hematopoietic cytokine produced by the fetal liver and adult kidney but, in the last decades, several studies support other roles in different tissues^23^. The brain^24^ and the retina^25^ were reported as new EPO secreting sites that also express EPO receptors^26,27^. A recent review article describes the antiapoptotic, angiogenic, anti-inflammatory, antioxidant and neuroprotective effects of EPO in the ocular tissues, including the retina^28^. Since EPOβ is a recombinant human EPO commonly used in medical practice, it was selected for this study with the perspective of contributing for glaucoma treatment as a neuroprotective agent.

Chitosan and hyaluronic acid are biocompatible polymers with notable mucoadhesive characteristics, that have a wide range of medical applications and have been extensively studied throughout these last decades for ophthalmological use^9–13,18–22^. Our team developed chitosan and hyaluronic acid (CS/HA) nanoparticles designed to carry EPOβ into the ocular environment, and described their physicochemical characteristics, in vitro safety and ex vivo permeation, amongst other features^4^. Nanoparticles’ size designed for ocular purposes ranges from 50 to 400 nm. Several studies using CS and HA nanoparticles support the optimal threshold for size as being 300 nm^11,21,29–32^. In a previous study, we managed to select the optimal nanoparticles formulation methods based on some trials, such as different CS:HA ratios, different solvents and different molecular weights for hyaluronic acid^4^. Therefore, we could not create nanoparticles much smaller than 300 nm without compromising their stability, zeta potential and polydispersity index. Our newly developed empty nanoparticles had 300 ± 6 nm, with a polydispersity index of 0.219 ± 0.043 and a zeta potential of 33 ± 1 mV; while nanoparticles loaded with 1000 IU of EPOβ presented a size of 289 ± 3 nm, polydispersity index of 0.126 ± 0.085, and zeta potential of 39 ± 1 mV^4^. Nanoparticles’ drug loading was 17.4 ± 0.1%, and the encapsulation efficiency was 38.4 ± 0.3%. In the in vitro release assay in simulated tear fluid (37 $^\circ$C, pH 7.4), 60% to 70% of EPOβ was released within the first 15 min, followed by a controlled release of nearly 90% of EPOβ in 6 h^4^. This means that EPOβ difuses from the CS/HA nanoparticles, whose role was to increase the corneal contact time by mucoadhesive properties of both polymers^21^, including HA binding to CD44 receptors in the corneal epithelium^20^. In addition, CS has the ability to open tight junctions between epithelial cells, allowing paracellular transport of large hydrophilic molecules^9^. These features contributed to the drug delivery enhancement following topical instillation assessed in the ex vivo assay. Ex vivo permeation results were promising, since the CS/HA nanoparticles allowed a considerably higher EPOβ permeation across the conjunctiva (up to 60% more), the sclera (up to 85.3% more) and the cornea (up to 2.5-fold more) compared to what was observed using NeoRecormon alone^4,33^. Moreover, CS/HA-EPOβ nanoparticles were noncytotoxic for ARPE-19 and HaCaT cells^4^. These promising results encouraged our team to proceed to the in vivo studies in Wistar Hannover (WH) rats, starting by assessing the effect of the CS/HA-EPOβ nanoparticulate system administered through the subconjuntival route, being able to conclude that the nanoformulation was biologically safe and enabled a sustained EPOβ retinal delivery for up to 21 days^5^. The topical route of administration was chosen for the following in vivo evaluation, because its non-invasive nature could represent a safe, low cost and high compliance therapeutical option using the CS/HA-EPOβ nanoformulation. Topical instillation of drugs targeting the posterior ocular segment is an expanding area of research, despite ocular mechanisms like blinking, nasolacrimal drainage and tear turnover, that hinder drugs permeation through ocular tissues. Topical ocular administration of nanocarriers might render neuroprotection accessible to a large number of patients suffering from vision threatening diseases caused by retinal degeneration.

In the present study, the size of the sample was n = 3 per each timepoint of euthanasia based on the power analysis using GraphPad StatMate 2 (GraphPad Software, CA, USA), sample size for unpaired t test, with a power of test of 80%, significance level of 0.05 and SD for ERG of 65 μV, which was estimated based on a previous study from our team^5^. Thus, for n = 3 we had 80% power to detect a difference between means in the ERG of approximately 189 μV with a significance level of 0.05 (two-tailed), which was considered adequate, also taking into consideration ethical issues of animal experimentation.

CS/HA-EPOβ nanoparticles were well tolerated by animals after their topical administration, as no signs of ocular lesions, discomfort or pain were observed, and animals presented normal behavior and normal ophthalmological examinations throughout the study. The average IOP measured immediately after the nanoparticles administration was little above the average for conscious WH rats, which is 18.4 ± 0.1 mm Hg^34^. This was attributed to ketamine effects, which is still present during the anesthesia recovery^35^. Despite the drop in IOP following the topical administration, values during the study were within the physiological range for both eyes*.* It is known that IOP fluctuates with the circadian cycle, and the reported average difference between the maximum and the minimum mean IOP for rats was 4.5 mmHg, and never exceeded 7.5 mmHg^36^. In the present study, the mean IOP difference in conscious rats was 5 mmHg, with no significant differences between OD and OS, which is in accordance with the reference values^36^. Probably, we could have avoided this variation if IOP measurements would have been performed at the same hour to produce less fluctuation in the IOP values. In addition, the fact that each group had 3 animals, contributed to the IOP variability amongst timepoints. No reports mentioning an eventual IOP lowering effect of EPO or EPOβ were found in the literature and no influence of CS or HA in the IOP after cataracts surgery was observed in a long-term clinical study^37^. Overall, these findings indicate that these nanocarriers are safe and well tolerated after topical ocular administration, which corroborate the results of our previous subconjunctival study^5^.

EPO stimulates erythropoiesis and the microhematocrit assessment was important to evaluate EPOβ’s eventual systemic side effects when administering CS/HA nanoparticles through the topical ocular route. No significant changes were observed in hematocrit values, which were always within the reference range for WH rats between 6 to 12 months of age^38^. Therefore, this nanoformulation appears to be systemically innocuous, which is in accordance with our previous results^5^.

One way to evaluate retinal physiology is using flash ERG, in which the retinal response to light stimulation is recorded as waveforms, representing the activity of different retinal cells^39^. The a-wave corresponds to the hyperpolarization of photoreceptors and it is the first negative deflection after the flash beginning, while the b-wave is a positive deflection that follows the a-wave and represents the activity of the bipolar and Müller cells^40^. In the photopic ERG, a-waves represent cone function, while in the scotopic ERG, a-waves indicate rod function^41^. Despite the different ERG protocols found in the literature, our results from the photopic and the scotopic ERGs performed in treated rats showed similar wave amplitudes and shapes to those recorded in control rats and also in previous studies using healthy Wistar Hannover rats^40,42^. The scotopic ERG results (SLR and SA) were comparable to the results of Bayer et al. (2001), using silver electrodes and the Ganzfeld stimulator in Wistar rats^43^. In that study, the b-wave amplitudes of the scotopic phase oscillated between 250 and 550 μV approximately^43^, which is within the same range of values recorded in our ERGs. On the other hand, the a-wave varied from nearly 110 μV to 150 μV^43^, which is in the mean range of both SA and SLR responses observed in our study. Considering the light-adapted ERG, the same study presents a b-wave between 200 and 250 μV^43^, which is comparable to our PA and PLR results. Moreover, the PLR results show that the b-wave increased with light intensity while the a-wave stayed nearly constant, which is understandable because when the light stimulus is excessive, the a-wave is not produced^44^. The PF results presented similarities to those found in the literature^3,42^, supporting that the cone function was maintained. Since no statistically significant changes in the ERGs waveforms or amplitudes were observed before and after topical administration of the CS/HA-EPOβ nanoparticles, and no differences were detected between OD and OS, it is reasonable to affirm that this nanoformulation did not influence retinal cells electric responses and, thus, retinal electrophysiology, which reinforces its local safety.

Immunofluorescence findings indicate that EPOβ was detected in all the treated eyes (OD, n = 18) and no EPOβ was observed in any of the control eyes (OS, n = 18). CS/HA nanoparticles topically administered efficiently delivered EPOβ to the retina. The fluorescent signal (EPOβ) was detected in the retinal ganglion cells of treated eyes (OD) 12 h after administration (Group A). Considering that EPOβ is highly soluble^45^, it could firstly follow the conjunctival-scleral route to the posterior ocular segment^46^, which justifies the presence of EPOβ in the retina only 12 h after its topical instillation*.* In the subconjunctival study, EPOβ was also observed in the retina 12 h after injection (Group A)^5^, which reinforces the hypothesis of the conjunctival-scleral absorption pathway. Likewise, in previous studies from our team using solely NeoRecormon, EPOβ was detected in the retina 12 h after its subconjunctival administration^47,48^. In this study, EPOβ was observed in the corneal stroma, corneal endothelium and ciliary body, suggesting that EPOβ diffused from the CS/HA nanoparticles and permeated the cornea as well, because the blood-aqueous barrier following the conjunctival-scleral pathway would prevent the presence of EPOβ in the anterior chamber^8^. Furthermore, EPOβ was still detected in the corneal stroma 14 days after the topical administration (Group E), indicating a sustained trans-corneal absorption, where nanoparticles persisted in the pre-corneal area and allowed a delayed EPOβ permeation. Trans-corneal permeation of EPOβ after topical administration of the CS/HA-EPOβ nanoparticles matches our previous ex vivo permeation results using porcine ocular membranes, which showed a slower EPOβ permeation across the cornea, when compared to the sclera and the conjunctiva^4^. In addition, our team had already proved the existence of a trans-corneal ex vivo permeation of EPOβ using solely a commercial solution (NeoRecormon)^33^. As EPOβ is negatively charged^49^, we did not expect a strong electrostatic binding within the vitreous, which is also negatively charged and normally acts as an important barrier to drug diffusion to the posterior segment^50^. Fluorescence declined with time and at day 21 there were still some fluorescent signals in the retina, meaning that 3 days of topical instillation of CS/HA-EPOβ were enough to deliver EPOβ to the retina for as long as 3 weeks. Although the in vitro release assessed previously showed that nearly 90% of EPOβ was released from the nanoparticles in 6 h^4^, these in vivo results indicate that CS/HA nanoparticles considerably increased EPOβ precorneal time, enabling its corneal permeation for, at least, 14 days, demonstrated by the presence of EPOβ in the corneal stroma at that timepoint. It seems that the mucoadhesive power of CS/HA nanoparticles was better appreciated in vivo. When comparing the grade of fluorescence with the subconjunctival study^5^, fewer fluorescent signals were observed in the ocular tissues using the topical route of administration, which is understandable considering the ocular barriers to overcome^51^.

HE staining revealed no cellular or structural modifications in any ocular globe, which reinforces the biological safety of the CS/HA-EPOβ nanoparticles at ocular level. This had already been demonstrated by both in vitro cytotoxicity assays^4^, and the previous in vivo subconjunctival study^5^.

We proposed to develop a patient-friendly drug delivery system of EPOβ to the retina using eyedrops with nanocarriers, which could be of value in the treatment of degenerative retinopathies. This nanoparticulate system based on CS/HA nanoparticles was effective in delivering EPOβ to the retina using the topical route of administration, allowing for a sustained EPOβ retinal delivery during 21 days. In vivo tests assessing the local and the systemic impact of this nanoformulation demonstrated that the CS/HA nanoparticles, with or without encapsulated EPOβ, were biologically safe, since no changes in ocular morphology or physiology were detected. The nanoformulation was very well tolerated by the animals after its topical instillation, which precludes a favorable patient’s acceptance as eyedrops.

The non-invasive route of administration, the high tolerance and the efficient EPOβ delivery to the retina render the CS/HA-EPOβ nanoparticulate system a promising formulation aiming for the neuroprotection in retinal diseases, such as glaucoma. Current glaucoma therapy is solely based on managing aqueous humor outflow and inflow, and it lacks a targeted and effective way to prevent visual loss. Neuroprotection is growing in the scope of scientific research and CS/HA-EPOβ nanoparticles as eyedrops represent a potential topical neuroprotective therapeutic option to preserve patient’s vision. Nevertheless, future research directions should include pharmacokinetic and pharmacodynamic studies of EPOβ after topical administration, and the perspective of testing this nanoformulation in an animal model of retinal disease.

## Material and methods

### Material

Animals used in this study were Wistar Hannover albino male rats (n = 18), weighting 330 ± 24 g, acquired from Charles River Laboratories (Saint-Germain-Nuelles, France). Ophthalmological equipment belonged to the Faculty of Veterinary Medicine (ULisboa), namely the slit lamp (Hawk Eye, Dioptrix, France), and the PanOptic ophthalmoscope (WelchAllyn—Hillrom, USA), the rebound tonometer (Tonolab, Icare, Finland) and the ERG device (RETIcom, Roland Consult, Stasche & Finger GmbH, Brandenburg, Germany). NeoRecormon 30,000 IU (RocheDiagnostics GmbH, Mannheim, Germany) was the Epoetin Beta (EPOβ) used in the nanoparticles. Low molecular weight Chitosan (LMW CS, 100 kDa, 92% deacetylation) was purchased from Sigma Aldrich (Irvin, UK). The eye grade quality Hyaluronic Acid, with an average Mw of 300 kDa, from Shandong Topscience, were a kind gift from Inquiaroma (Barcelona, Spain). Ketamine (Ketamidor 100 mg/mL, Richter Pharma, Wels, Austria) and medetomidine (Domtor 1 mg/mL, Orion Corporation, Espoo, Finland) were used to anesthetize the animals, and atipamezole (Antisedan 5 mg/mL, Zoetis, New Jersey, USA) was used to revert anesthesia. For euthanasia, sodium pentobarbital (Euthasol 400 mg/mL, Animalcare Group, North Yorkshire, UK) was used. All these drugs were available at the Faculty of Veterinary Medicine (ULisboa). Epredia SuperFrost Plus Adhesion slides (ThermoFisher Scientific, Massachusetts, USA) were used in immunofluorescence assays. The cover plates, the immunostaining rack (Epredia Shandon Sequenza, ThermoFisher Scientific, Massachusetts, USA) and the slide stainer for hematoxylin and eosin stain (Thermo Scientific Gemini AS, Massachusetts, USA) belonged to the Faculty of Veterinary Medicine (ULisboa). HepG2 human derived liver hepatocellular carcinoma cell line (ATCC HB-8065) was used as immunofluorescence control. All cell culture media and supplements were from Gibco (ThermoFisher Scientific, Massachusetts, USA). EPO monoclonal primary antibody 4F11 (MA5-15684) and goat anti-mouse IgG (H + L) secondary antibody DyLight 488 (35502) were from Invitrogen (ThermoFisher Scientific, Massachusetts, USA). The blocking reagent (sc-516214) and the mounting medium with DAPI (sc-2494) were from UltraCruz (Santa Cruz Biotechnology, Texas, USA). Axioscop 40 fluorescence microscope with an Axiocam HRc camera (Carl Zeiss, Germany) belonged to the Faculty of Pharmacy (ULisboa).

### Methods

#### Animals

Animals used in this study were Wistar Hannover male rats (n = 18) which were randomly split into 6 groups of 3 animals each. Animals were housed in type IV cages (1875 cm^2^ of floor area) with a stainless-steel wire cover, one group (n = 3) per cage, with food pellets and water ad libitum*.* The room was maintained in a 12-h light/ darkness cycle and the temperature (20 ± 2 °C) and the humidity (50–60%) were constantly controlled. Timepoints were selected after the topical administration of CS/HA-EPOβ nanoparticles to perform euthanasia, as follows: 12 h (group A), 1 day (group B), 3 days (group C), 7 days (group D), 14 days (group E) and 21 days (group F). This study was performed according to the ARRIVE and the Declaration of Helsinki guidelines and also to the Portuguese and European Union legislation concerning animal welfare (DL 113/2013 and Directive 2010/63/UE). It was approved by the Ethical Committee for Research and Education and by the Organ Responsible for Animal Welfare (Órgão Responsável pelo Bem-Estar dos Animais—ORBEA) of the Faculty of Veterinary Medicine, University of Lisbon, approval date February 13, 2020, code 005/ 2020; and by the national entity General Directorate of Food and Veterinary (Direção Geral de Alimentação e Veterinária—DGAV), approval date January 8, 2021, code 0421/ 000/ 000/ 2020.

#### Ophthalmological examination

A complete ophthalmological examination was performed in every animal before the study, which included biomicroscopic examination of the anterior segment with a Slit Lamp (Hawk Eye, Dioptrix, France), posterior segment examination with a PanOptic ophthalmoscope (WelchAllyn—Hillrom, USA) and the measurement of the intraocular pressure (IOP) with a Rebound Tonometer (Tonolab, Icare, Finland). Complete ophthalmological examinations were also performed 1 h, 12 h and 1, 2, 3, 7, 14 and 21 days after the topical administration of CS/HA-EPOβ nanoparticles.

#### Preparation of nanoparticles

Nanoparticles were prepared before the first electroretinography. Reagents were previously sterilized by filtration in a laminar flow cabinet using a 0.22 µm filter. Nanoparticles were also prepared in a laminar flow cabinet, to maintain their sterility, by a modified ionotropic gelation technique described in a previously published protocol developed by our group^4,21,22^. To the chitosan solution at 1 mg/mL in NaCl 0.9% was added 1000 IU of EPOβ (NeoRecormon) in hyaluronic acid solution (1 mg/mL). The empty CS/HA nanoparticles (without EPOβ), which were used in the negative controls, were prepared following the same protocol, by adding purified water instead of EPOβ. Both the CS/HA-EPOβ and the CS/HA formulations were aspirated with sterilized insulin syringes and kept at room temperature until the topical administration.

#### Electroretinography and Hematocrit measurement

General anesthesia with 70 mg/kg of ketamine and 0.8 mg/kg of medetomidine was administered intraperitoneally and a blood sample was collected from a lateral vein of the tail with a capillary tube. After being centrifuged at 10,000 rpm for 5 min, hematocrit was read using a proper scale. This was performed before the first and also before the second electroretinography (ERG). A heating pad was used to prevent hypothermia and the animal’s body temperature was periodically checked. One drop of oxybuprocaine hydrochloride (Anestocil, Edol, Carnaxide, Portugal) and one drop of a carbomer based gel (Lubrithal, Dechra Pharmaceuticals PLC, Northwich, United Kingdom) was instilled on both corneas, and an active electrode with a silver tip was placed on each eye. Reference electrodes (blue) were placed subcutaneously on both sides of the head, so that the tip of the electrode was located between the ear and lateral canthus. At the base of the tail, a ground electrode (black) was placed and a MiniGanzfeld was the light-stimulation device that was adjusted to the animal’s head. All animals underwent an ERG test before the administration of CS/HA-EPOβ nanoparticles, to assess the previous status of the retina, and before euthanasia, to evaluate the outcome of the nanoparticles’ topical administration. The ERG protocol was adapted from a previously published method^40^, and a scotopic adaptation of 12 h before the ERG exam was mandatory. The exam was split in five steps, with a total duration of 75 min, organized as follows: 1st scotopic luminance response (SLR); 2nd photopic adaptation (PA); 3rd photopic luminance response (PLR); 4rd photopic flicker (PF) and 5th scotopic adaptation (SA). ERG results were independent for each eye, but they were recorded simultaneously. The anesthesia was then reverted with atipamezole (2.5 mg/kg) through intramuscular administration.

#### Topical administration of nanoparticles

Topical ocular administration of the nanoparticles was initiated immediately after the first ERG, at a rate of one drop every 5 min, until a total amount of 80 µL/eye (6 drops in average). The right eye (OD) received CS/HA-EPOβ nanoparticles corresponding to 1000 IU of EPOβ. The left eye (OS) received empty CS/HA nanoparticles (without EPOβ) and represented the negative control.

#### Euthanasia and enucleation

At 12 h (group A), 1 day (group B), 3 days (group C), 7 days (group D), 14 days (group E) and 21 days (group F) after the administration of the CS/HA-EPOβ formulation, euthanasia was executed in all animals of each group, immediately after the second ERG, by intraperitoneal injection of sodium pentobarbital (150 mg/kg). Both ocular globes were enucleated right after the euthanasia and the optic nerve and the lateral, medial, dorsal, and ventral sides were painted with tissue dyes to facilitate orientation for paraffin inclusion and histological sections. Ocular globes were preserved in 10% (v/v) formaldehyde in PBS (0.1 M, pH 7.4) and then processed to be included in paraffin blocks.

#### Histological assessment

Immunofluorescence and hematoxylin and eosin (HE) staining were performed in both ocular globes of all animals. After paraffin inclusion, eight cross sections (3 μm) per eye were made using a microtome. Four sections were placed in adhesion slides for immunofluorescence and four sections were placed in regular slides for HE staining, being these last ones processed in the multi-tasking stainer Gemini AS. The immunofluorescence slides were deparaffinized in xylol and gradually rehydrated in alcohol and purified water, followed by washing steps with Triton X-100 and Tween 20 solutions. Simultaneously, HepG2 cells (positive control) were previously in a hypoxic environment for 2 h at 37 °C and were fixed in 10% (v/v) formaldehyde in PBS, washed with Triton X-100 solution and then followed the same protocol as the cross sections. Slides were assembled in cover plates and incubated with UltraCruz Blocking Reagent for one hour at room temperature, followed by incubation with EPO monoclonal primary antibody 4F11 (1:400), overnight at 4 °C. After washing with Tween 20 solution, incubation with goat anti-mouse secondary antibody DyLight 488 (1:1000) was performed in the dark, for one hour, at room temperature. Slides were cautiously disassembled from the cover plates, assembled with UltraCruz mounting medium with DAPI and with a coverslip, being afterwards sealed with varnish. The analysis of the sections was performed using an Axioscop 40 fluorescence microscope with an Axiocam HRc camera (Carl Zeiss, Germany), in which the recorded images were processed using AxioVision software (Rel.4.8.1, Carl Zeiss). The observed EPOβ fluorescence was green (OD), the negative control was the OS and the positive control, to ensure the quality of the immunofluorescence technique, were HepG2 cells.

#### Statistical methods

Statistical assessment was performed with GraphPad Prism version 6.0 (GraphPad Software, CA, USA) and Microsoft Office Excel (Microsoft, Washington, USA), using one-way ANOVA and t-test to detect significant differences between means. The statistical significance was 95%, which corresponds to a *p*-value of 0.05, data being presented as mean ± standard deviation (SD).

### Ethical statement

The study was conducted according to the ARRIVE and the Declaration of Helsinki guidelines and also to the Portuguese and European Union legislation concerning animal welfare (DL 113/2013 and Directive 2010/63/UE). The study was also approved by the Ethical Committee for Research and Education and by the Organ Responsible for Animal Welfare (Órgão Responsável pelo Bem-Estar dos Animais—ORBEA) of the Faculty of Veterinary Medicine, University of Lisbon approval date 13 February 2020, code 005/2020, and the national entity General Directorate of Food and Veterinary (Direção Geral de Alimentação e Veterinária—DGAV) approval date 8 January 2021, code 0421/000/000/2020. No informed consent was obtained since animals used was laboratory species.

## Data Availability

The authors confirm that the data supporting the findings of this study are available within the article.
